# Solid Dispersions as a Tool for Innovation in the Food Industry: A Path From Pharma to Food

**DOI:** 10.1111/1750-3841.70917

**Published:** 2026-02-18

**Authors:** Stephany C. de Rezende, Arantzazu Santamaria‐Echart, Madalena M. Dias, Maria Filomena Barreiro

**Affiliations:** ^1^ CIMO, LA SusTEC Instituto Politécnico de Bragança, Campus de Santa Apolónia Bragança Portugal; ^2^ LSRE‐LCM, ALiCE, Faculty of Engineering University of Porto, Rua Dr. Roberto Frias Porto Portugal

**Keywords:** food innovation, food products, nutraceuticals, pharmaceutical, solid dispersion

## Abstract

Solid dispersion (SD) is a technique used to improve the solubility of poorly water‐soluble compounds by dispersing them in a solid water‐friendly carrier. Current trends indicate that natural‐based alternatives are increasingly replacing synthetic carriers, benefiting the pharmaceutical industry, where they were first adopted, and paving the way for broader use in nutraceuticals and food applications, as regulations and consumer preferences drive the adoption of eco‐friendly alternatives. In the food industry, SDs can address key challenges, such as enhancing water compatibility and stabilizing sensitive compounds, thereby facilitating the effective use of natural‐based ingredients. Exploring natural carriers enables SDs to align with food industry priorities, enabling the development of functional ingredients, stable natural colorants, products with increased flavor retention, innovative packaging materials, and healthier, structured food analogues through Pickering emulsion technology. In this context, the review examines the path of SDs from pharma to food, beginning with a detailed examination of SD systems using both synthetic and natural carriers across the pharmaceutical, nutraceutical, and food sectors. The review concludes with an in‐depth discussion of emerging applications in the food industry, highlighting the potential of SDs to address formulation challenges and to foster sustainable, consumer‐oriented innovations in modern food systems. To advance SD applications in food systems, future research should integrate sensory evaluation and address technical, regulatory, and formulation‐performance gaps to ensure consumer‐acceptable, high‐quality innovations.

## 1 Introduction to Solid Dispersions

Among the various strategies designed to enhance the bioavailability of hydrophobic compounds, SD is a preferred methodology in the pharmaceutical industry. Since its introduction in the 1960s (Nair et al. 2020; Sekiguchi and Obi 1961) and particularly named solid dispersion (SD) in the 1970s (Chiou and Riegelman 1971), SD has been widely recognized as a valuable approach for developing stable and efficient drug formulations, leading to continuous evolution and significant advancements in the field (Khan et al. 2022).

SD involves the dispersion of at least two components, commonly a hydrophobic crystalline active compound and a hydrophilic carrier, typically a polymer, where the active compound is molecularly dispersed within the polymer matrix, resulting in an amorphous structure (Mir and Khan 2017; De Mohac et al. 2020; Pasarkar et al. 2022). The chemical composition and processing techniques are crucial for designing a successful SD formulation, which can result in different structures stabilized by intermolecular interactions. SDs are commonly classified in different generations, reflecting advancements in the field since the first SD was developed in 1961 (Sekiguchi and Obi 1961). This first‐generation utilizes a crystalline carrier, producing eutectic mixtures where the melting point is lower than that of the active compound and the carrier (Tekade and Yadav 2020). The second generation introduces amorphous carriers, improving the active compound dissolution rate compared to the first generation. The major challenges associated with these systems are precipitation under supersaturation and recrystallization of the active compound (Sanklecha 2020). Third‐generation SDs, which have generated significant interest over the last decade, utilize carriers with emulsifying properties or a mixture of amorphous polymers and emulsifiers. This advancement addresses the disadvantages of the second generation, further enhancing the dissolution rate of the active compound and improving its physical and chemical stability (Tambosi et al. 2018; Budiman, Lailasari, et al. 2023).

The fourth generation comprises controlled‐release systems of active compounds with short half‐lives, using hydrophobic or swellable polymers to slow the release (Srividya and Ghosh 2025). Recent research has suggested a fifth generation involving multicomponent SDs. These systems, designed to further enhance SD performance, consist of one or more hydrophobic active compounds dispersed within a carrier that comprises more than two polymers (De Mohac et al. 2020).

Another classification categorizes SDs by dispersion type. It comprises binary SDs, ternary SDs, and solid surface dispersions. Binary SDs are a dual‐phase system, including a hydrophobic active compound and a hydrophilic carrier. Ternary SDs comprise three components, corresponding to the addition of a surfactant to the active compound and carrier (Saberi et al. 2023). Solid surface dispersion is a system where the active compound is selectively deposited onto the carrier surface. This configuration often yields smaller particle sizes, thereby enhancing dissolution rates and bioavailability (Sanklecha 2020).

SDs offer higher solubility and dissolution rates, as well as improved stability of hydrophobic active compounds, making them appealing systems for drug formulation (Huang and Dai 2014). This technology can be applied to various compounds, enhancing their performance while offering effective, continuous, scalable production opportunities. In this context, SDs are widely used at an industrial scale for their process efficiency and the convenience of producing a final product in powder form, with their small particle sizes also contributing to promoting dissolution and increasing absorption rates (Joy et al. 2020; P. Tran et al. 2019).

Maintaining the chemical and physical stability of SDs during storage can be challenging. In fact, the literature frequently highlights the limitations of carrier hydrophilic polymers, as they often absorb moisture, reversing the transition from the amorphous to the crystalline state and thereby decreasing solubility and dissolution rates (Dhande et al. 2021; Cid et al. 2019). Thus, it is essential to properly select the chemical systems used and assess their stability during production and storage.

The evolution of SD carriers began with the use of synthetic polymers, such as polyvinylpyrrolidone (PVP) and polyethylene glycol (PEG), which offered improved stability and controlled release. Further advancements led to the use of cellulose derivatives and enteric polymers to enhance the molecular interaction between the active compound and the polymeric carrier. The progress then faced a growing preference for natural, biocompatible polymers such as chitosan and cyclodextrins, which offered enhanced safety and compatibility (S. Jain et al. 2024).

Building on this trend, significant effort has been dedicated to understanding and refining SD methodology to achieve optimal formulations for practical applications (Wang et al. 2023). The pharmaceutical industry, known for its pioneering innovations, has been a primary adopter, commercializing numerous products that use SDs to improve drug efficacy. Thereafter, as the pharmaceutical sector advanced, SD steadily expanded into other areas. Recognizing the potential of SD to address solubility, bioaccessibility (the fraction of a bioactive compound available for absorption), and bioavailability (the amount effectively absorbed to reach the site of action), areas such as cosmetics, nutraceuticals, and food began adopting this approach. For example, in the nutraceutical field, SD has helped to improve the absorption and effectiveness of dietary supplements (Colombo et al. 2021). Similarly, SD has been applied to develop functional food ingredients with enhanced properties (Tomas et al. 2024).

This expanding use of SD beyond the pharmaceutical sector highlights its versatility and effectiveness in addressing formulation challenges across diverse sectors. It reflects a continuous effort to innovate and apply scientific advancements to meet the evolving needs of different industries. In line with this, the present work offers an overview of the transition of SD from pharmaceutical to food applications. It reviews recent research and key developments in this field, paving the way for this transition.

## 2 Chemical Systems and the Role of Molecular Interactions

This section provides a concise overview of the key components used in SD systems, including carriers, active compounds, and surfactants. In this context, the materials with higher potential for SD carriers can be categorized into polymeric materials (PVP, polyvinyl alcohol [PVA], PEG, cellulose derivatives, gums, cyclodextrin, pectin); sugars (dextrose, lactose, maltose, mannitol, sorbitol, sucrose, xylitol); and miscellaneous (urea, pentaerythritol, hydroxyalkyl xanthene) (Ghule et al. 2018; Sharma et al. 2019). In the case of polymeric carriers, which are widely used, selection is crucial, as it influences SD features such as active compound release kinetics, physical stability, and mechanical properties. The hydrophilicity of polymers, particularly in amphiphiles, can enhance particle wettability, thereby improving water solubility (Kaushik et al. 2020). Depending on the specific SD application, several factors must be considered, including the polymer's ability to effectively entrap the active compound and its solubility in commonly used solvents. Moreover, they must exhibit a high glass transition temperature (*T*_g_) to ensure physical stability during storage, while also possessing suitable thermal and rheological properties at processing temperatures (e.g., low melt viscosity or softening behavior) to facilitate melting, dissolution, and efficient drying (Rusdin et al. 2024).

The active ingredients, which are poorly soluble in water, must be compatible with the carrier to avoid low polymer‐active compound interactions. Most advancements concerning active compounds have focused on conventional drugs, with several SD systems in commercialization in the pharmaceutical market (Pandi et al. 2020). Examples include lopinavir/ritonavir (used in the treatment of HIV/AIDS) (Trasi et al. 2019), itraconazole (antifungal) (Chivate et al. 2021), fenofibrate (used in the treatment of high cholesterol and triglyceride levels) (Choudhary et al. 2018), and celecoxib (anti‐inflammatory) (Chen et al. 2015).

Surfactants, or surface‐active agents, are used in SDs to enhance the dissolution rate of active compounds and improve their physical stability (Chaudhari and Dugar 2017). Surfactants can also aid in uniformly dispersing the active compound throughout the carrier material. The surfactant must be miscible with the carrier polymer to be effective and cannot cause any instability during storage (e.g., crystallization) (Solanki et al. 2019). Surfactants may also be added post‐production, resulting in a significant positive effect on the release of the active compound (França et al. 2018). Examples of surfactants include Tween, Span, sodium lauryl sulfate, gelucire, poloxamer, polyglycerol esters of fatty acids, and sucrose esters of fatty acids.

**FIGURE 1:**
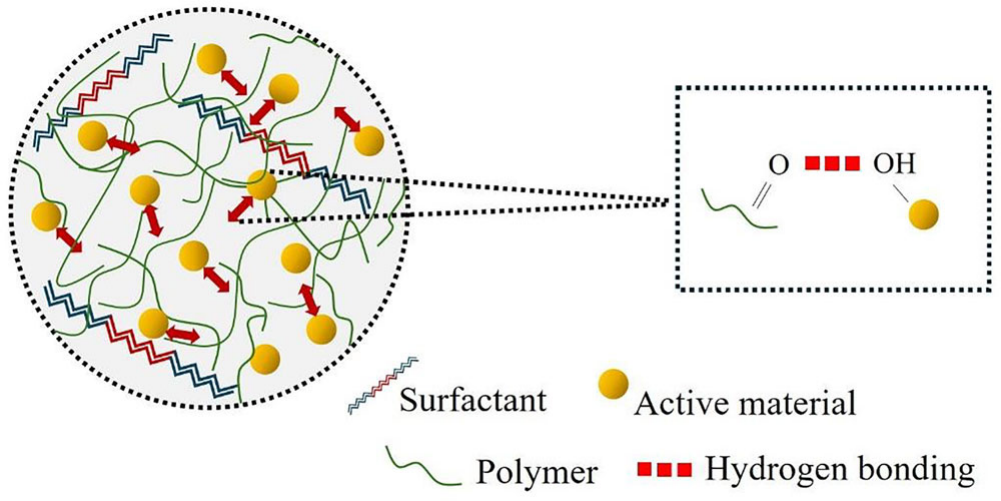
Schematic illustration of a solid dispersion particle. *Source*: Figure adapted from Hanada et al. (2023).

In summary, the selection of components significantly influences the formation and performance of SDs. In fact, the dissolution and solubility of the active ingredients are conditioned by their interaction with the polymer and other excipients present in the formulation, which typically restricts the active compounds’ crystallization, the main purpose of the SD technology. To this, two principal mechanisms are recognized: (i) the “spring,” a supersaturated dissolution of the active compound in the solution medium, derived from its amorphization, leading to higher and more rapid solubilization compared to the crystalline form, and (ii) the “parachute,” the supersaturation stabilization effect led by the applied polymer, responsible for hindering the recrystallization of the active compound, while maintaining the nano/microentities in a dissolved state in the solution medium (liquid–liquid phase separation) (Ramachandran et al. 2025; Kawakami 2025). Understanding the molecular interactions developed between the active compound and the carrier is crucial for predicting solubility, stability, and the release mechanisms of the entrapped compounds (Tran and Tran 2020). The importance of molecular interactions in the physical stability of SDs is well‐documented and widely studied. Specifically, active compounds containing polar functional groups, such as hydroxyl or carbonyl groups, can form hydrogen bonds with hydrophilic carriers containing complementary polar moieties (as illustrated in Figure 1). These hydrogen bonds restrict molecular mobility, thereby hindering the nucleation and crystal growth of the active compound. This mechanistic interaction contributes directly to the formation and physical stabilization of SDs (Kothari et al. 2015; Colombo et al. 2021).

Depending on the chemical nature of the carrier and the active compound, additional molecular interactions may be formed. For example, ionic interactions can occur when ionizable groups are present, thereby promoting stronger attraction between SD components and enhancing molecular compatibility within the polymer matrix (T. T. D. Tran and Tran 2020). Hydrophobic interactions may be relevant in systems containing nonpolar or amphiphilic carriers, contributing to the stabilization of the amorphous structure through interactions between hydrophobic regions. These interactions should be considered when designing SD systems (Ramachandran et al. 2025).

Recent studies have increasingly focused on examining the associations between polymers and hydrophobic active compounds to better understand their role in SDs’ performance and stability (Lu et al. 2019; Tran and Tran 2020; Yang et al. 2020). For example, the interactions involving curcumin have been studied with PVP, poloxamer 188, and hydroxypropyl‐β‐cyclodextrin (HP‐β‐CD), with PVP giving the most promising results in terms of solubility and stability, considering the stronger active compound‐polymer interactions formed (He et al. 2019). The hydrogen bonding between the enolic or phenolic hydroxyl groups of curcumin and the carbonyl groups of PVP was identified as the predominant interaction. Other research on curcumin‐based SDs (Luu et al. 2019), which utilized modified sprouted rice as the carrier, reported the formation of hydrogen bonding between curcumin and the carrier, involving the enolic and phenolic hydroxyl groups of curcumin and the hydroxyl or carboxyl groups of the sprouted rice.

Recognizing the importance of molecular interactions in determining the behavior and stability of SDs, molecular simulations using Hansen solubility parameters and hydrogen‐bond formation energies have been used to predict miscibility and interactions between the active compound and the carrier (Yani et al. 2017). This work clarified the interactions between active compounds and commonly used polymers, providing predictions of hydrogen‐bonding energies and their stabilities. Validation via SDs preparation confirmed the stabilizing influence of hydrogen bonds across diverse systems, emphasizing their crucial role in achieving a stable molecular mixing rather than mere physical mixing.

## 3 Overview of Preparation Methods

The physicochemical properties of SDs are influenced by the preparation conditions, which are critical for promoting hydrogen bonding interactions and allowing the transition of the active compound from a crystalline to an amorphous state. SDs can be produced using different methodologies, namely, the melting or fusion method, the solvent evaporation method, and the hybrid melting‐solvent method (Al‐japairai et al. 2023). Among these, the melting method stands out as the most straightforward approach; however, it has drawbacks, such as component degradation at high temperatures (Meng et al. 2015) or induction to secondary reactions (i.e., Maillard browning reaction) whose presence can condition the attributes of the product, including color, taste, or generation of toxic compounds (Xiang et al. 2021).

The solvent evaporation method mitigates these issues using lower temperatures (Budiman, Lailasari, et al. 2023). Still, it becomes challenging to eliminate the organic solvent, which may introduce instability to the systems. The melting‐solvent method aims to combine the advantages of both methods; however, it is restricted by the amount of active compound that can be used (Mallick et al. 2020; Nikam et al. 2020).

In the melting or fusion method, dispersion can be achieved through simple melting, hot‐melt extrusion, or melt agglomeration. In simple melting, the active compound and the carrier are mixed at or above the respective melting points. The mixture is then cooled or frozen using various methods, such as spreading it on a chilled stainless‐steel plate, using cold air, an ice bath, or immersing it in liquid nitrogen, then grinding to achieve a powder form (Kaushik et al. 2020). In hot‐melt extrusion, the active compound and the carrier are pre‐mixed before being subjected to hot fusion. This method is similar to the simple melting technique but involves vigorous mixing at high rotation speeds in an extruder. Depending on the application, the final product is collected as granules, tablets, or pellets (Manogna et al. 2017). Melt agglomeration is described as a technique in which the polymer acts as a binder that can be melted. The agglomeration can be processed in different ways: either by adding the carrier and active compound mixture to excipients in a rotating processor or by incorporating the melted carrier into a mixture containing the active compound and excipients. In addition, the different ingredients can be heated above the binder's melting point, and the resulting agglomerates can be cooled using methods similar to those described for simple melting, such as cold air, ice baths, or cooled surfaces, and then processed into a powder (Bindhani and Mohapatra 2018).

In solvent evaporation techniques, the dispersion step involves dissolving and mixing the polymer and active compound in a common solvent or, when they are incompatible, in two miscible solvents (such as water and ethanol), followed by solvent evaporation. This stage can be achieved through vacuum drying, rotary evaporation, and spray drying, which involve removing the solvent by heat (Budiman, Handini, et al. 2023). Among the three, spray drying is a continuous and scalable method for producing micro‐ to nano‐sized particles, which may be advantageous for subsequent applications. This technique is very attractive for preparing amorphous SDs, as it enables rapid solvent evaporation, increasing viscosity, and facilitating the kinetic trapping of the active compound within the carrier matrix, resulting in a supersaturated molecular dispersion (Dedroog et al. 2022). The fluid is fed to the drying chamber, where atomization occurs through an atomizer or a two‐fluid nozzle driven by centrifugal, pressure, or kinetic forces. When exposed to the drying gas, the resulting droplets experience rapid solvent evaporation, forming the dry particles. Finally, a cyclone is used to separate the particles from the drying gas (Singh and Van den Mooter 2016). An alternative to the described techniques, which is not strictly a solvent‐evaporation method, comprises freeze‐drying (sublimation) to remove the solvent, yielding a dry molecular dispersion that can be further reduced to a powder (Bashir Mir and Ahmed Khan 2017). If applied, the organic solvent must be removed before the dispersion is freeze‐dried.

Some patented techniques have also been reported, such as Kinetisol Dispersing, developed to produce SDs based on high fusion energy using high shear mixing combined with high temperature (Prasad et al. 2016), and Meltrex, which uses a special twin‐screw extruder together with two self‐governing hoppers in which temperature varies over a wide range (Kaushik et al. 2020). Less commonly used methods include the supercritical anti‐solvent (SAS) technique. In this approach, the active compound and carrier are dissolved in supercritical CO_2_, and the solution is sprayed through a nozzle into an expansion chamber at lower pressure, forming the SD particles (Abuzar et al. 2018).

## 4 Solid Dispersions Evolution Through Time

**FIGURE 2:**
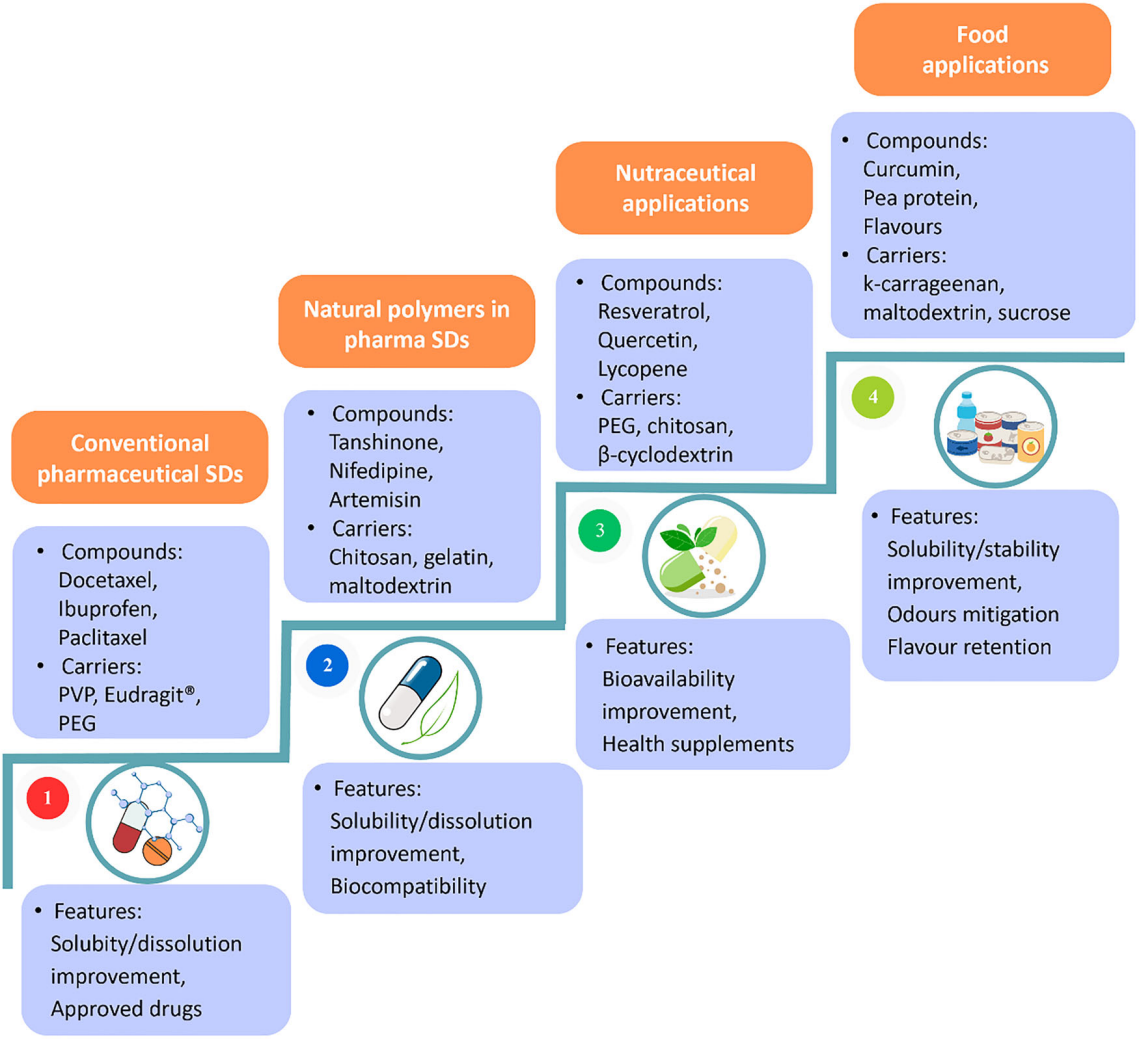
Evolution of solid dispersion technology from conventional pharmaceutical systems to food applications, highlighting technological advancements grouped into four key stages. PEG, polyethylene glycol; PVP, polyvinylpyrrolidone; SD, solid dispersion.

Over the years, considerable efforts have been made to better understand SD technology and develop optimal formulations. The pharmaceutical area was a pioneer in this field, accounting for a greater number of commercialized products based on SDs. Parallel to the evolution of this principal market, SD technology started to be applied to other sectors, namely, the cosmetic, nutraceutical, and food sectors. In this context, Figure 2 illustrates the progressive evolution of SD applications, structured into four main stages.

The first stage represents the conventional pharmaceutical SDs, primarily focused on improving the solubility and dissolution rate of poorly water‐soluble drugs using synthetic hydrophilic carriers such as PVP, PEG, and Eudragit. In the second stage, a shift toward safer, biodegradable, and more biocompatible alternatives leads to the incorporation of natural polymers, such as chitosan, gelatine, and maltodextrin, thereby enhancing solubility and biological compatibility. The third stage marks the expansion of SD technology into the nutraceutical sector, where it is utilized to improve the bioavailability of bioactive compounds, including resveratrol, quercetin, and lycopene (Tomar et al. 2022). This trend aligns with growing consumer demand for natural health supplements with improved efficacy. The final and most recent stage reflects the integration of SDs into the food sector, with a focus on natural compounds such as curcumin, flavors, and plant proteins. In this context, SDs enhance solubility and stability, while also enabling odor masking and flavor retention, key functionalities that support clean label initiatives and the development of innovative functional food products.

## 5 Pharmaceutical Area: From Synthetic to Natural Carriers

A substantial body of literature exists on the use of SD technology in the pharmaceutical sector, with major companies such as Hovione, Johnson & Johnson, and Lilly implementing it in their preparation methods (Ganesan et al. 2015; Lino and Henriques 2019). Between 2010 and 2020, the US Food and Drug Administration (FDA) approved over 20 such products in the pharmaceutical sector (Zhang, Guo, et al. 2023; Bhujbal et al. 2021). Initial products used synthetic carriers (Table S1), namely, PVP and derivatives such as polyvinylpyrrolidone/vinyl acetate (PVPVA), polymethacrylate derivatives (Eudragit), polyvinyl caprolactam–polyvinyl acetate–PEG grafted copolymer (Soluplus), hydroxypropyl methylcellulose (HPMC), hydroxypropyl methylcellulose acetate succinate (HPMCAS), and PEG. The widespread use of PVP is due to its high solubility in water and organic solvents, its capacity to form a stable amorphous matrix with drugs, and its role in improving bioavailability (Rusdin et al. 2024). Among the drugs tested with PVP are docetaxel, paclitaxel, bosentan, raloxifene hydrochloride, azithromycin, and nifedipine, resulting in improved solubility and dissolution rates (Sawicki et al. 2016; Harish et al. 2017; Elkanayati et al. 2024; Huynh et al. 2023) and optimal stability during storage (Saraf et al. 2022).

Although its prominence has declined, PVP remains important, as illustrated by indomethacin‐based SDs prepared with PVP and HPMC (Jarrells et al. 2025; Martynek et al. 2025) and the use of the copolymer PVPVA to encapsulate nifedipine, providing good performance, as indicated by a single *T*_g_, which reflects physical stability and homogeneous phase mixing (O'Connell et al. 2025). Other examples include apremilast and GDC‐0334 incorporated into PVPVA‐based SDs (L. Yang et al. 2021; Chiang et al. 2023) and naproxen in Kollidon VA 64, a branded PVPVA from BASF (Kissi et al. 2021).

Polymers with registered trademarks were also extensively used. This is the case of Eudragit and Soluplus (Lin et al. 2018; Biedrzycka and Marcinkowska 2023; Metre et al. 2018; Aldeeb et al. 2023; Harish et al. 2017; H. Wang et al. 2022). Eudragit offers multiple grades for targeted drug release in the gastrointestinal tract sites, and Soluplus, a thermoplastic polymer developed for hot‐melt extrusion, is well‐suited for advanced applications (Giri et al. 2021; A. Nikam et al. 2023). Other examples include Kollicoat Smartseal, two methacrylate‐based formulations, and Pluronic F127, a block copolymer of polyethylene oxide and polypropylene oxide with amphiphilic properties (Chivate et al. 2021; Agafonov et al. 2021). HPMC and HPMCAS are also considered suitable choices for applications involving controlled drug release, with HPMCAS being particularly effective in delayed‐release or enteric formulations (Smeets et al. 2018; Mora‐Castaño et al. 2024).

Overall, SDs based on synthetic carriers improve solubility and dissolution, overcoming challenges with hydrophobic drugs and boosting oral bioavailability. Research shows their solubility far exceeds that of physical mixtures (Shamsuddin et al. 2016). However, the commercial trend toward natural‐based products has spurred research into developing natural‐based equivalents (Table S2). Natural polymers are a viable alternative to synthetic polymers due to their broad applicability, nontoxicity, and affordability (S. Jain et al. 2024). In this context, recent studies on SDs have explored various natural polymers, including carbohydrates, natural gums and their derivatives, proteins, and others.

Maltodextrin has been used as a carrier in SDs containing the antifungal drug nystatin, significantly enhancing its anti‐biofilm activity compared to the pure drug (Benavent et al. 2021). Moreover, when combined with Arabic gum in SDs, it successfully preserved artemisinin's potent antimalarial efficacy (Meliana et al. 2020). Other examples include gums, namely, novel gums like *Ziziphus spina‐christi* gum, used to prepare SDs for diverse drugs (loratadine, glimepiride, and furosemide) (Alwossabi et al. 2022). Additionally, Arabic, guar, xanthan, and locust bean gums were explored as carriers for carvedilol, a medication used for heart failure and hypertension, and etoricoxib, an analgesic and anti‐inflammatory agent (Sopyan et al. 2023; Sapkal et al. 2020).

Although the aforementioned gums improved solubility, SDs prepared with sodium alginate showed better results (Sopyan et al. 2023). Namely, studies using alginate with dexlansoprazole and tanshinone have significantly improved drug bioavailability (Gulia et al. 2023; Luo et al. 2020), with biosafety and biocompatibility advantages. Chitosan was also used for its ability to enhance drug wettability (Grimling et al. 2014), with successful examples including andrographolide and tanshinone (Luo et al. 2019; Sari et al. 2019). A comparative study of SDs of diflunisal with chitosan revealed that formulations prepared by solvent evaporation and vacuum drying outperformed those prepared by the kneading method in terms of dissolution rate (Lucio et al. 2022).

Natural proteins have also been explored as carriers in recent studies. Examples include bovine serum albumin, egg white protein, gelatin, and zein (Khoder et al. 2018; Telange et al. 2021; Pas et al. 2018; V. Ngo et al. 2016). Among these, whey protein isolate showed better performance overall or as individual fractions, such as α‐lactalbumin, casein glycomacropeptides, and β‐lactoglobulin (Leng et al. 2023; Mishra et al. 2019; Zhuo et al. 2023). Other less commonly used natural polymers include sugars such as isomalt, maltitol, and saccharin, as well as porous starch derived from mung beans (França et al. 2022; Nadaf et al. 2021).

Overall, it must be emphasized that using natural polymers not only promotes sustainability goals but also complies with regulatory approval and consumer expectations for safer excipients (Umekar et al. 2025; Mahesha et al. 2025). They also corroborate the use of natural polymers as versatile carriers that enhance solubility, ensure physical stability, and enable effective control of drug release (Budiman et al. 2025). This emphasis on natural‐based formulations, driven by customer and environmental needs, has also influenced related fields, promoting a “greener” trend in the nutraceutical area.

## 6 Nutraceutical Area: Bridging Pharmaceuticals and Foods

Nutraceuticals, which combine nutritional and medicinal properties, have garnered significant interest in recent years due to their health benefits (Daliu et al. 2018). Alongside, the food industry has focused on developing innovative products incorporating these compounds. Despite their potential, many nutraceuticals face challenges, including low water solubility, bioavailability, and stability (Tomar et al. 2022). Among various strategies to overcome these limitations, SD technology stands out as a promising solution. This technology has effectively enhanced the solubility, absorption, and efficacy of a range of active compounds, including resveratrol, vitamin D, apigenin, curcumin, lycopene, and some fatty acids. While some studies continue to develop SD formulations using conventional synthetic polymers, the prevailing trend is to meet market demand by producing SDs entirely from natural ingredients. Recent studies have further advanced SD formulations by introducing novel natural carrier‐nutraceutical combinations, confirming the ongoing innovation in this field (J. Zhang et al. 2025; Alali et al. 2025). Table 1 presents examples of SD technology in nutraceuticals, using both synthetic and natural carriers.

**TABLE 1:** Solid dispersions prepared with synthetic and natural carriers for the nutraceutical area, highlighting the type of carrier, active ingredient, preparation method, and principal results.

Synthetic carriers				
Carrier	Active compound	Preparation method	Main results	Reference
Eudragit	Resveratrol	Spray drying	SD improved the solubility, maintaining the supersaturated state for 48 h with no resveratrol precipitation	Ha et al. (2021)
Eudragit E PO	*Centella asiatica* extract	Solvent evaporation—vacuum drying	SD significantly enhanced the solubility and sustained release of the glycosides asiaticoside and madecassoside	Wannasarit et al. (2020)
Eudragit E PO	Resveratrol	Freeze‐drying	SD increased resveratrol solubility by 8 to 12 times, improved intestinal permeability, and showed a 5 times higher dissolution rate compared to the pure form and the physical mixture	Almeida et al. (2024)
Eudragit E PO, PEG 6000, Kollidon 30 (PVP K30), and Soluplus	Resveratrol	Solvent evaporation—room temperature	SD with PVP K30 and Soluplus showed better miscibility and stability but low dissolution rate, while Eudragit E PO improved dissolution about 13 times without changing crystallinity or stability after storage	Yu et al. (2023)
HPMCAS, HPMCP, Soluplus, cellulose acetate, and Gelucire 50/13	Epigallocatechin gallate	Freeze‐drying	The physical stability and dissolution rate, especially with Soluplus, were improved	Cao, Teng, and Selbo (2017)
Hydroxypropyl cellulose‐SSL	Nobiletin	Freeze‐drying	SD improved the solubility and oral bioavailability	Nihei et al. (2021)
PEG 6000 and F68	Resveratrol	Melting method	SD raised the solubility and dissolution rate significantly	L. Wang et al. (2021)
PEGs 4000 and 6000	*Moringa oleifera* leaf powder	Freeze‐drying, melting, solvent evaporation—oven drying, and microwave irradiation	SDs of Moringa extract (partially amorphous state) provided better thermal stability than the pure compound	Tafu and Jideani (2021)
Pluronic F127	Apigenin	Spray drying	SD at a 1:4 (apigenin:Pluronic F127) ratio significantly improved dissolution rate and bioavailability (through indicated hydrogen bonding between the components)	Altamimi et al. (2018)
Pluronic F127	Apigenin	Kneading, melting, and microwave irradiation	SD improved the dissolution rate and oral absorption	Alshehri et al. (2019)
PVP K10	Resveratrol	Solvent evaporation—vacuum drying	SDs revealed stable amorphous or partially crystalline systems, with molecular‐level distribution and hydrogen bonding networks that prevent recrystallization	Pajzderska et al. (2025)
PVP K30 and Eudragit E PO	Ginger extract	Solvent evaporation—vacuum drying	SD increased the solubility of the ginger extract. Raft‐forming system included sodium alginate and HPMC	Matchimabura et al. (2024)
PVP K30 and poloxamer	Vitamin D3	Solvent evaporation—room temperature	SD improved cholecalciferol solubility, maintained its amorphous form and stability, showed no adverse effects on intestinal cells, and enhanced the dissolution rate of HPMC capsules	Rawat et al. (2023)
PVP K30 and PVPVA64	Pterostilbene	Dry ball milling	SDs improved the solubility, release profile, permeability, antioxidant properties, and neuroprotective effects of pterostilbene	Rosiak et al. (2024)
PVP K30	Myricetin	Spray drying	The solubility of myricetin was improved up to 50% of the nutraceutical load	Mureşan‐Pop et al. (2017)
PVPVA64 and Soluplus	Piperine	Freeze‐drying, solvent evaporation—oven drying, and microwave irradiation	SDs prepared with PVPVA64 and Soluplus improved solubility and stability, with ternary systems showing superior performance over binary ones	Imam et al. (2025)

Natural carriers				
Carrier	Active compound	Manufacture technique	Main results	Reference
Arabinogalactan	Curcumin	Dry ball milling	SD significantly increased curcumin's solubility, stability, and bioavailability	Q. Zhang et al. (2019)
Chitosan	Abietic acid	Solvent evaporation—agitation at room temperature	SD exhibited enhanced antioxidant and antimicrobial properties (particularly at 1:1 abietic acid:chitosan ratio)	Cuzzucoli Crucitti et al. (2018)
Chitosan	Apigenin	Spray drying	SD showed enhanced drug release, improved antimicrobial and antioxidant properties, and potential anticancer effects	Alali et al. (2025)
Chitosan	Quercetin	Dry ball milling	SD enhanced the dissolution rate and increased 2.25 times the bioavailability compared with pure quercetin	Han et al. (2021)
Erythritol	Oleocanthal	Melting method	SD increased the dissolution rate, improving the nutraceutical properties of oleocanthal	Tajmim et al. (2021)
Microcrystalline cellulose	*Moringa oleifera* leaf extract	Freeze‐drying	SD improved the solubility and physical‐mechanical characteristics of moringa extract (1:2 ratio moringa:cellulose ratio)	Rani et al. (2023)
Myricetin (co‐former)	Curcumin	Freeze‐drying	SD improved curcumin dissolution in intestinal fluid and its bioavailability, with enhanced antioxidant activity	J. Zhang et al. (2025)
Modified sprouted rice	Curcumin	Melting method	SD enhanced the dissolution rate of curcumin	Luu et al. (2019)
*Undaria pinnatifida* polysaccharides	Ellagic acid	Dry ball milling	SD significantly enhanced ellagic acid's solubility, dispersibility, and biotransformation efficiency and improved microbial accessibility	Li et al. (2023)
Xylitol	Oleocanthal	Melting method	SD enhanced the dissolution rate and effective taste masking, potentiating in vivo anti‐breast cancer activity	Qusa et al. (2019)
α‐Glycosylated stevia	Sesamin	Spray drying	SD increased the solubility and bioavailability, improving the potential of the nutraceutical properties	Sato et al. (2017)
β‐Cyclodextrin	Lycopene	Freeze‐drying	SD exhibited promising solubility for osmotic‐controlled delivery (1:5 lycopene:𝛽‐cyclodextrin ratio)	N. Jain et al. (2014)
β‐Cyclodextrin	Lycopene	Solvent evaporation—vacuum followed by oven drying	SD improved the thermal, light stability and antioxidant activity of lycopene	H. Wang et al. (2019)

Synthetic/Natural carriers				
Carrier	Active compound	Manufacture technique	Main results	Reference
PVP K30 and mannitol	Ellagic acid	Solvent evaporation—vacuum drying	SDs enhanced the solubility and stability of ellagic acid under stress conditions	Kawoosa et al. (2025)
Sodium alginate, Pluronic F‐68, Pluronic F‐127, PVP K30, and PVPVA64	Apigenin	Dry ball milling	SD, particularly Pluronic F‐127‐based, significantly improved apigenin's solubility, stability, and antioxidant activity	Rosiak et al. (2025)

*Note*: In the table, “manufacture technique” primarily refers to the final drying step in solvent‐evaporation methods for SD, particularly spray drying or freeze‐drying. However, these processes are preceded by essential steps, including the dissolution and mixing of components in a common or miscible solvent, as detailed in Section 3.

Abbreviations: HPMCAS, hydroxypropyl methylcellulose acetate succinate; HPMCP, hypromellose phthalate; PEG, polyethylene glycol; PVP, polyvinylpyrrolidone; PVPVA, polyvinylpyrrolidone/vinyl acetate.

Nutraceutical SDs produced with synthetic carriers employ the same conventional polymers utilized in pharmaceutical formulations, including PVP, Eudragit, Soluplus, HPMC, HPMCAS, and PEGs, as detailed in Section 3. Resveratrol, a natural polyphenolic compound found in grapes, berries, and peanuts, is renowned for its antioxidant, anti‐inflammatory, and potential anti‐cancer properties. However, its clinical use is hindered by poor water solubility and rapid metabolism, resulting in low bioavailability (Biagini et al. 2024). Studies indicate that the solubility, dissolution rate, and oral and gastrointestinal absorption of resveratrol can be significantly improved when combined with Eudragit, resulting in enhanced therapeutic efficacy and more consistent pharmacokinetic profiles (Almeida et al. 2024; Ha et al. 2021; Yu et al. 2023). In addition, recent work has demonstrated that resveratrol SDs using PVP K10 exhibit stable amorphous or partially crystalline forms, in which hydrogen‐bonding interactions effectively prevent recrystallization, thereby improving solubility and ensuring long‐term stability of formulations (Pajzderska et al. 2025). Eudragit has also been employed in SDs containing *Centella asiatica* extract, yielding exceptional solubility profiles for asiaticoside and madecassoside, as well as for triterpenoid saponins present in the extract (Wannasarit et al. 2020). SDs of piperine, prepared with a combination of PVPVA64 and Soluplus as carriers, further demonstrated that ternary systems can outperform binary ones in terms of solubility and stability, highlighting the advantage of synthetic polymer mixtures in enhancing SD performance (Imam et al. 2025).

Vitamin D3, another active compound exhibiting poor water solubility, was incorporated into PVP K30 and poloxamer SDs coated with HPMC, resulting in improved dissolution rate and stability, thereby enhancing oral bioavailability (Rawat et al. 2023). Similar to its role in pharmaceuticals, PVP exhibits remarkable efficacy in improving the solubility of nutraceuticals. Examples include ginger extract, pterostilbene (a resveratrol derivative), and myricetin (Matchimabura et al. 2024; Rosiak et al. 2024; Mureşan‐Pop et al. 2017), where the bioavailability increased by 50% of the total load in the case of myricetin (Mureşan‐Pop et al. 2017). Besides its role as the active compound, myricetin has also been used as a co‐former in a drug‐drug co‐amorphous SD with curcumin, where hydrogen bonding between the two compounds improved curcumin dissolution and bioavailability compared to the pure form (J. Zhang et al. 2025).

Apigenin, a naturally occurring plant flavonoid known for its health benefits, such as antioxidant, anti‐inflammatory, anticancer, and neuroprotective activities, was subjected to SD technology using Pluronic F127 (poloxamer F127), which improved its bioavailability, thereby increasing its effectiveness in nutraceutical applications (Altamimi et al. 2018; Alshehri et al. 2019). Comparative evaluations confirmed that Pluronic F127 outperformed other carriers in enhancing apigenin's stability and antioxidant activity (Rosiak et al. 2025). PEGs, including PEG F68, PEG 6000, and PEG 4000, have also been applied, significantly enhancing the solubility and stability of compounds such as resveratrol and *Moringa oleifera* leaf powder (L. Wang et al. 2021; Tafu and Jideani 2021). Additionally, hydroxypropyl cellulose, HPMCP, and HPMCAS have been studied in formulations containing epigallocatechin gallate (a major catechin in green tea) and nobiletin (a flavonoid derived from citrus peels) (Cao et al. 2017; Nihei et al. 2021). Although promising results were obtained regarding dissolution rate and stability, Soluplus outperformed various polymers, including HPMCAS and HPMCP (Cao et al. 2017).

Research on natural polymers in nutraceutical applications has driven notable progress, as highlighted in recent reviews that list numerous studies using various active compounds (Mohapatra et al. 2021; Colombo et al. 2021; Tomar et al. 2022). Curcumin, derived from turmeric, has potent antioxidant and anti‐inflammatory properties. Widely regarded as a nutraceutical, curcumin has substantial therapeutic potential for various health conditions and is associated with improved joint, heart, and digestive health. The innovative use of SD technology significantly enhances its effectiveness, strengthening its role as a vital component of overall well‐being (Khursheed et al. 2022). In a recent study, curcumin SDs were produced with arabinogalactan, a biopolymer consisting of arabinose and galactose, as the carrier. The SD reduced curcumin's crystallinity, enhancing solubility by approximately 10.5 times, while also demonstrating high chemical stability and improved membrane permeability. Pharmacokinetic studies in rats showed an 8‐fold increase in bioavailability compared with pure curcumin (Q. Zhang et al. 2019). Similar to curcumin, lycopene, a potent carotenoid predominantly found in tomatoes and other red fruits, known for its high antioxidant and anti‐inflammatory activities, markedly increased its bioavailability when incorporated into SDs. Furthermore, it has been shown to increase its health benefits, including lowering cancer risk, improving cardiovascular health by reducing LDL cholesterol levels, and providing protection against UV‐induced skin damage (Wu et al. 2024). Recent studies on lycopene SD formulations, primarily using β‐cyclodextrin as a carrier, have demonstrated an impressive 18‐fold increase in water solubility, along with significant improvements in thermal resistance and photostability (N. Jain et al. 2014; H. Wang et al. 2019).

Another example involves oleocanthal (an active compound found in olive oil) and xylitol (a carrier), which were used to produce SDs through the melting technique. The optimized formulation not only improved the dissolution rate of the active compound but also effectively masked undesirable flavors (Qusa et al. 2019). Similarly, oleocanthal was explored using erythritol, a sugar alcohol used as a low‐calorie sweetener, as the carrier. The resulting SD formulations significantly enhanced the dissolution rate of oleocanthal and improved memory deficits in the 5xFAD mouse model, highlighting its promising potential in controlling Alzheimer's disease progression (Tajmim et al. 2021).

As already highlighted in Section 3.2, chitosan is a natural carrier widely applied in the nutraceutical and pharmaceutical fields. When used in SDs, chitosan significantly enhances the solubility of abietic acid, a tricyclic diterpene with anti‐inflammatory, antimicrobial, and antioxidant properties, as well as that of quercetin, a flavonoid known for its diverse biological activities. This improvement effectively leveraged their bioactivity, demonstrating remarkable results in optimizing their therapeutic potential (Cuzzucoli Crucitti et al. 2018; Han et al. 2021). Building on these findings, recent studies have confirmed the versatility of chitosan, showing that SDs of apigenin enhance its antimicrobial, antioxidant, and anticancer potential (Alali et al. 2025).

Microcrystalline cellulose, *Undaria pinnatifida* polysaccharides, and α‐glycosylated stevia are natural carriers that have been recently investigated for their bio‐based nature and potential to develop SD nutraceutical formulations. These polymers are being investigated for their potential to enhance and prolong the effectiveness of SD formulations, offering promising opportunities to develop advanced, efficient nutraceutical products (Rani et al. 2023; Li et al. 2023; Sato et al. 2017). Other strategies have also been explored, including hybrid systems that combine synthetic and natural carriers, as exemplified by ellagic acid SDs with PVP K30 and mannitol, as well as comparative studies assessing the performance of synthetic versus natural carriers, such as the case of apigenin SDs, to identify formulations with higher efficacy (Kawoosa et al. 2025; Rosiak et al. 2025).

Despite the advances, the scientific community continues to make significant strides in refining formulations. Efforts are focused on developing more efficient delivery systems to expand the application and efficacy of nutraceutical products. Moreover, researchers are exploring the potential of SD technology within the food sector, expanding beyond nutraceutical applications to create more effective and innovative solution products.

## 7 Food Area: Emerging Applications

In recent years, SD technology has advanced into the food sector, emphasizing the use of natural polymers, driven mainly by the need to meet legislation requirements. In this context, the effectiveness of SDs can overcome the limitations of several food ingredients, improve their water solubility, mitigate off‐flavors, provide stable colors, and enhance water compatibility for natural‐based colorants (Lan et al. 2019; Cui et al. 2020). Examples of how SD technology is overcoming these and other limitations are provided in Table 2. It is evident that although a few studies on SDs for food applications still rely on synthetic polymers, most solutions emphasize natural polymers to align with industry demands for more sustainable formulations. In the case of natural‐based solutions, comparisons with well‐established synthetic polymers, such as PVP or HPMC, serve as benchmarks, allowing a direct evaluation of systems developed with natural polymers and thereby highlighting the current research focus.

**TABLE 2:** Solid dispersions for the food area, highlighting the type of carrier, active ingredient, preparation method, and principal results.

Carrier	Active compound	Preparation method	Main results	Reference
Casein	Palm kernel stearin and tristearin	Freeze‐drying	SDs were successfully developed using palm kernel stearin, whereas tristearin exhibited partial crystallinity, highlighting the potential of SDs as functional food ingredients with improved aqueous dispersibility	Vyas and Harte (2025)
Cyclodextrin	Pea protein isolate	Spray drying	SD mitigated the beany odor, maintaining the functional properties of the active compound	Cui et al. (2020)
Gum Arabic and maltodextrin	Pea protein isolate	Spray drying	SD increased the solubility, and beany flavors were mitigated	Lan et al. (2019)
HPMCAS‐HF and sorbitan monolaurate	Lycopene	Solvent evaporation—oven drying	SDs showed full amorphization, improved antioxidant activity, high biocompatibility, and protective effects in a liver injury model	Su et al. (2025)
HPMC, lecithin, and isomalt	Curcumin	Hot melt extrusion	SD showed 13 times higher bioavailability and enhanced anti‐inflammatory effects compared to raw curcumin, providing a formulation with improved sensory properties for functional food ingredients	Chuah et al. (2014)
*κ*‐Carrageenan	Curcumin	Freeze‐drying	SDs were uniformly dispersed in *κ*‐carrageenan films, enhancing mechanical properties and offering antioxidant, antimicrobial, and nonmigratory protection for olive oil	Rezende, Santamaria‐Echart et al. (2024)
*κ*‐Carrageenan	Curcumin	Spray drying	SDs were employed as innovative Pickering stabilizers, producing emulsions with light mayonnaise‐like properties, extended shelf life, and enhanced health and functionality	Ghirro et al. (2022)
Pectin	Curcumin	Freeze‐drying	SDs enabled the production of stable Pickering emulsions with tailored texture and color properties	Rezende et al. (2025)
PVP and sucrose fatty acid ester	Beta carotene	Melting technique	SD led to the amorphization of beta carotene improving its solubility	Ishimoto et al. (2019)
PVP and sucrose fatty acid ester	Beta carotene	Hot melt extrusion	SD increased the water dissolution ratio by reducing the base materials:active compound ratio	Ishimoto et al. (2021)
PVP and sucrose fatty acid ester	Beta carotene	Hot melt extrusion	SD improved the oral bioavailability in rats	Otani et al. (2020)
PVP, *κ*‐carrageenan, maltodextrin, Arabic gum, potato starch, and pectin	Curcumin	Freeze‐drying	SD significantly enhanced curcumin's water solubility. The natural polymers showed good performance compared to the synthetic PVP	Rezende, Ferreira et al. (2024)
PVP, PVA, and *κ*‐carrageenan	Curcumin	Spray drying	SD resulted water‐dispersible evidencing pH and heat stability	Leimann et al. (2019)
PVP K30	Hexahydrocolupulone	Solvent evaporation—vacuum drying	SD increased the solubility and presented promising antibacterial and antioxidant activities. The addition of SD improved the sensory and nutritional quality and microbiological properties of fresh apple juice	Zhang, Liu, et al. (2023)
PVP/Disaccharides (α‐maltose, palatinose, sucrose, and trehalose) (amorphized)	Fat‐soluble flavors: cinnamaldehyde, anethole, citral, eugenol, carvacrol, and raspberry ketone	Solvent evaporation—vacuum‐foam‐drying and spray drying	SD produced through vacuum‐foam‐drying with PVP, α‐maltose, or palatinose presented enhanced flavor retention results than other methodologies (e.g., traditional O/W emulsification method of powderization)	Nitta et al. (2024)
Steviol glycoside	Phloretin	Solvent evaporation—vacuum drying	SD enhanced the solubility of the active compound and showed a higher dissolution rate compared with the micelles’ preparation approach	F. Wang et al. (2020)
Sucrose, α‐maltose, trehalose, α‐lactose, and maltitol (amorphized)	Fat‐soluble flavors: cinnamaldehyde, anethole, citral, ethylvanillin, eugenol, and raspberry ketone	Solvent evaporation—vacuum drying	SD presented better flavor retention results than other methodologies (e.g., traditional O/W emulsification method of powderization)	Satoh et al. (2016)

*Note*: In the table, “manufacture technique” primarily refers to the final drying step of solid dispersion in solvent evaporation methods, particularly spray drying or freeze‐drying. However, these processes are preceded by essential steps, including the dissolution and mixing of components in a common or miscible solvent, as detailed in Section 3.

Various strategies have been developed to encapsulate hydrophobic substances, thereby overcoming the constraints to their application in the food industry. These include conventional micro to nano‐emulsions, Pickering emulsions, liposomes, solid lipid particles, nanostructured lipid carriers, hydrogel systems, and complexation techniques (Premathilaka et al. 2022). Colloidal systems, such as micro‐ to nano‐emulsions, are beneficial due to their nontoxic nature, the absence of organic solvents, and their ability to provide prolonged release of hydrophobic substances. However, their high costs and reliance on large quantities of surfactants, which are often replaced with natural‐based alternatives, also limit their use (Hosseini et al. 2021).

Recent research emphasizes the effectiveness of SDs in enhancing flavor retention compared to traditional methods. One study has shown that embedding hydrophobic flavoring substances in PVP and disaccharides significantly minimized flavor loss during drying and storage (Nitta et al. 2024). These findings were reinforced by Satoh and co‐workers, who revealed that SD using amorphous disaccharides retained 65%–95% of the flavors of cinnamaldehyde, anethole, citral, ethylvanillin, eugenol, and raspberry ketone, whereas their incorporation into a traditional oil‐in‐water (O/W) emulsion led to substantial loss (Satoh et al. 2016). These studies suggest that SD is a superior approach for preserving flavors.

Various vegetable‐extracted proteins and carotenoids face significant application challenges in the food industry due to their unpleasant odors and low water solubility. Traditional encapsulation methods may lead to protein denaturation, with some studies suggesting reduced solubility (Lan et al. 2019). Alternatively, recent studies have highlighted formulations on the basis of SDs; for example, pea protein isolate has been effectively formulated with carriers such as gum Arabic and maltodextrin (Cui et al. 2020) and cyclodextrin (Lan et al. 2019), resulting in improved solubility, flavor mitigation, and maintenance of functionality.

Lipophilic triglycerides such as palm kernel stearin and tristearin, formulated with casein, have also been studied. Particularly, palm kernel stearin achieved complete amorphization, while tristearin retained partial crystallinity, highlighting both the potential and limitations of protein carriers for highly hydrophobic lipids in food applications (Vyas and Harte 2025). Additionally, hexahydrocolupulone, a hop extract component, and phloretin, a polyphenol predominantly found in apple tree bark, were successfully formulated with PVP and steviol glycoside as carriers, respectively. Both studies focused on enhancing the water solubility of these active compounds (Zhang, Liu, et al. 2023; F. Wang et al. 2020).

Carotenoids, hydrophobic compounds highly valued for their nutritional and coloring properties, are known for their stability problems. Traditional encapsulation techniques, such as solid lipid particles, offer solutions but also pose limitations, including legislative constraints, excipient restrictions, limited active compound loading capacity, and the release of active compounds during storage (Paliwal et al. 2020). In this context, the need to find solutions drives the development of advanced encapsulation techniques and the adoption of effective techniques from other industries. An example includes the use of SDs to improve the water solubility of beta‐carotene and curcumin (Ishimoto et al. 2019; 2021; Otani et al. 2020; Chuah et al. 2014). Moreover, β‐carotene, curcumin, and lycopene SDs produced with HPMCAS‐HF, a high‐pH soluble HPMCAS form, and sorbitan monolaurate showed enhanced antioxidant activity and biocompatibility, and in vivo tests indicated a reduction of oxidative stress and liver damage (Su et al. 2025). From a different perspective, SD technology has opened new avenues for developing effective natural colorant solutions, an emerging field with significant potential. Recent works have addressed this theme by presenting water‐dispersible curcumin‐based SD colorant systems for food applications (Leimann et al. 2019; Rezende, Ferreira, et al. 2024). Using natural‐based polymers, SD offers a robust solution to improve carotenoid solubility and create stable, natural colorants for the food industry.

Another innovative application is the integration of SD formulations into polymeric films for food packaging. Starch films reinforced with curcumin SDs, produced using the natural carrier steviol glycoside, have shown enhanced biological and physical properties (F. Wang et al. 2023). Recently, *κ*‐carrageenan films functionalized with curcumin *κ*‐carrageenan SDs were developed to overcome the challenge of incorporating hydrophobic functionalities into hydrophilic matrices. These films exhibited enhanced mechanical properties and potent antioxidant and antimicrobial activities and offered a nonmigratory active protection mechanism for olive oil matrices (Rezende, Santamaria‐Echart, et al. 2024). Building on this progression, the innovative use of curcumin SDs produced from *κ*‐carrageenan as Pickering stabilizers was a significant breakthrough in the field. It is known that Pickering emulsions, namely, emulsions stabilized by solid particles, have great potential, as their stabilizing mechanism, typically a physical barrier formed by the particles at the oil and water interface, provides them with enhanced stability compared with conventional surfactant‐based emulsions (Santamaria‐Echart et al. 2021). According to this recently published study, the produced Pickering emulsions can mimic commercial products, presenting an attractive bright yellow color, acidic pH, and texture similar to light mayonnaise, with the advantages of improving oxidative stability and extending shelf life, while serving as healthy, functional food alternatives (Ghirro et al. 2022). Following the same approach, pectin‐based particles have also effectively stabilized Pickering emulsions, combining natural polymer functionality with curcumin SDs to achieve stable, food‐grade emulsions with controlled droplet size, desirable texture, and enhanced shelf life (Rezende et al. 2025). In fact, by modulating the formulation of Pickering emulsions (i.e., particle concentration and oil fraction), their rheology, color, and applicability can be tailored, ranging from gel‐like emulsions suitable for sauces or spreads to fluid emulsions for dressings or beverages.

These advancements highlight the versatility and transformative potential of SD technology, shaping the development of functional and sustainable packaging solutions and driving innovation in food products.

## 8 Challenges and Future Work

Although SD is an established technique in the pharmaceutical field, its application in foods still presents significant challenges, particularly in ensuring compatibility with food‐grade requirements while meeting cost, sensory, and clean‐label expectations. The use of natural polymers as alternative carriers offers promising potential for addressing these challenges, although their validation under realistic food processing and storage conditions remains limited.

Beyond carrier selection, recent research is exploring green processing technologies to enhance SD sustainability and performance in food systems. Among them, supercritical fluid‐based approaches, particularly those using supercritical CO_2_, are being used. Supercritical CO_2_ combines gas‐like diffusivity with liquid‐like density, enabling efficient mass transfer at relatively low temperatures while significantly reducing or eliminating the use of organic solvents (Uwineza and Waśkiewicz 2020). Processing routes such as SAS and rapid expansion of supercritical solutions (RESSs) enable the formation of fine particles with controlled morphology and improved dispersion of active compounds in the polymeric carriers (Kumar et al. 2021). These techniques are especially suitable for thermosensitive and hydrophobic compounds, having demonstrated to enhance solubility and physical stability while lowering solvent consumption and environmental impact in SD systems (Mahesha et al. 2025). The combination of supercritical CO_2_ with natural polymers can be a step forward in developing greener, more sustainable alternatives for the food industry.

SD's physical stability is also challenging, as moisture uptake, phase separation, and recrystallization can compromise the amorphous state and reduce efficacy. Consequently, developing predictive stability models that accurately reflect food‐relevant conditions, along with accelerated tests that reliably correlate with shelf life, remains critical for successful product development. Ultimately, regulatory approval will be crucial for advancing SD applications in food products. Although natural polymers already used in the food industry generally meet safety standards, regulatory guidance may still be necessary to support new SD applications, namely, regarding particle size and morphology, which, below certain limiting sizes and characteristics, can be linked to toxicity risks. One advantage is that SDs form through physical assembly (no chemical reaction involved), which is beneficial for digestibility purposes and reduces toxicity risks. This process maintains the native structure of food‐grade carriers and avoids covalent modifications that could hinder enzymatic degradation or impact safety. However, their digestibility must still be carefully evaluated.

Future research should also prioritize sensory evaluation, as SD characteristics can influence taste, texture, and visual appearance. Systematic sensory studies are crucial for providing insight into consumer perception, supporting the development of formulations with consistent quality and acceptable sensory performance. To this end, the design of appropriate SD preparation methodologies and the processing of SD‐loaded final food matrices must be carefully controlled to avoid undesirable reactions (viz., Maillard browning or caramelization), which can directly affect the color, taste, and even the nutritional profile of the final products.

Overall, advancing the field of SDs requires addressing both technical, including sensorial, and regulatory challenges, as well as clarifying the relationships between formulation strategies and functional performance in real food systems. By bridging these gaps, SDs can progress beyond proof‐of‐concept studies, facilitating broader application and the development of innovations that are effective and meet consumer expectations.

## 9 Conclusions

SDs are an effective strategy for enhancing the solubility and bioavailability of hydrophobic compounds. Over time, SD formulations and preparation methods have evolved from early drug‐focused approaches to broader applications, increasingly emphasizing green chemistry by replacing synthetic carriers with natural ones, particularly natural polymers. Widely established in the pharmaceutical industry, SD technology is now positioned for growth in the food industry. Its potential to improve solubility and stability and to address challenges such as protein off‐flavors and colorant stabilization makes it especially promising for functional foods. The ongoing shift toward natural, sustainable materials aligns with regulatory trends and consumer demands, positioning SDs as a key strategy for advancing both food innovation and environmental sustainability.

## Author Contributions

**Stephany C. de Rezende**: conceptualization, methodology, investigation, writing – original draft. **Arantzazu Santamaria‐Echart**: conceptualization, supervision, writing – review and editing. **Madalena M. Dias**: supervision, writing – review and editing. **Maria Filomena Barreiro**: conceptualization, supervision, resources, funding acquisition, writing – review and editing.

## Conflicts of Interest

The authors declare no conflicts of interest.

## Supporting information




**Supplementary Material**: jfds70917‐sup‐0001‐SuppMat.docx

## Data Availability

The authors have nothing to report.
