# Supplementary material for: Solid Dispersions as a Tool for Innovation in the Food Industry: A Path From Pharma to Food
Source: J Food Sci. 2026 Feb 18;91(2):e70917. doi: 10.1111/1750-3841.70917 (PMC12917352; doi:10.1111/1750-3841.70917)
Supplement: Supplementary file 1 — Supplementary Material: jfds70917‐sup‐0001‐SuppMat.docx [file JFDS-91-0-s001.docx]

**Supplementary material**

**for**

**Solid dispersions as a tool for innovation in the food industry: A path from pharma to food**

Stephany C. de Rezende^1,2^, Arantzazu Santamaria-Echart^1,*^, Madalena M. Dias^2^ and Maria Filomena Barreiro^1,*^

*^1^ CIMO, LA SusTEC, Instituto Politécnico de Bragança, Campus de Santa Apolónia, 5300-253 Bragança, Portugal*

*^2^ LSRE-LCM, ALiCE, Faculty of Engineering, University of Porto, Rua Dr. Roberto Frias, 4200-465 Porto, Portugal.*

*Corresponding author. E-mail address: A. Santamaria-Echart ([asantamaria@ipb.pt](mailto:asantamaria@ipb.pt)), M.F. Barreiro ([barreiro@ipb.pt](mailto:barreiro@ipb.pt))

Table S1. Pharmaceutical solid dispersions prepared with synthetic polymers, highlighting the carrier, active ingredient, preparation method, and principal results.

| **Carrier** | **Active compound** | **Preparation method** | **Main results** | **Reference** |
| --- | --- | --- | --- | --- |
| D-α-tocopherol PEG 1000 succinate (TPGS) and PVPVA | Apremilast | Spray drying | Enhanced dissolution rate and *in vivo* oral bioavailability. | (L. Yang et al. 2021) |
| Eudragit® E PO | Ibuprofen, felodipine and bifendate | Hot melt extrusion | Enhanced solubility for the three active compounds. | (Lin et al. 2018) |
| Eudragit® E PO | Ibuprofen | Hot melt extrusion | Amorphous and stable SD for three months. Best dissolution rate achieved for formulations using extrusion temperature of 140 ºC. | (Biedrzycka and Marcinkowska 2023) |
| Eudragit® S100, Eudragit® L100 and Soluplus® | Rivaroxaban | Spray drying | Improved solubility and intestinal uptake. | (Metre et al. 2018) |
| Eudragit®, PVP and PEG 4000 | Mirtazapine | Solvent evaporation - vacuum oven drying | Enhanced dissolution rate and solubility. Oral bioavailability 1.34 times higher compared to the plain drug. | (Aldeeb et al. 2023) |
| HPMC | Felodipine | Hot melt extrusion | Enabled ideal drug release kinetics for a gastroretentive system. Allowed for a 3D-printed pharmaceutical tablets formulation. | (Mora-Castaño et al. 2024) |
| HPMC, HPMCAS and PVP | Darunavir | Spray drying and electrospraying | Enhanced dissolution rate for all the carriers. | (Smeets et al. 2018) |
| Kollicoat® Smartseal | Itraconazole | Hot melt extrusion | Solubility improved 20 times compared to the pure drug. | (Chivate et al. 2021) |
| Kollidon® VA 64 | Naproxen | Hot melt extrusion | The amorphous SD remained stable in 3D-printed tablets. | (Kissi et al. 2021) |
| PEG 4000 | Atorvastatin calcium | Melting technique | Enhanced solubility and dissolution rate. | (Shamsuddin et al. 2016) |
| Pluronic® F127 | Methotrexate | Melting technique | Increased oral bioavailability. | (Agafonov, Ivanov, and Terekhova 2021) |
| Poloxamer 188 | Paclitaxel | Spray drying | Enhanced dissolution rate and oral bioavailability. | (Liu et al. 2024) |
| PVP | Docetaxel and paclitaxel | Spray drying | Completely amorphization of the active compounds. Improved solubility compared to freeze-dried SDs. | (Sawicki et al. 2016) |
| PVP  and polyethylene oxide | Raloxifene hydrochloride | Hot melt extrusion | Improved active compound release for the formulation containing a higher amount of PVP and tween 80 (optimised). | (Elkanayati et al. 2024) |
| PVP K12 | Indomethacin  and indomethacin methyl ester | Cryogenic ball milling and melting technique | Drug stability in SDs depended on drug load, storage temperature relative to Tg, and hydrogen bonding. Optimal conditions (low drug load and storage below Tg) allowed to inhibit crystallization | (Jarrells, Yuan, and Munson 2025) |
| PVP K30 and HPMC E5 | Indomethacin | Spray drying and hot melt extrusion | Hot-melt extrusion yielded more stable indomethacin SDs (especially with PVP K30). Spray drying gave higher dissolution but lower stability. | (Martynek et al. 2025) |
| PVP K30 and K90 | Nifedipine | Dry ball milling | Drug more stable in SDs with longer-chain PVP K90 than with PVP K30. | (Saraf et al. 2022) |
| PVPVA and HPMCAS | GDC-0334 | Spray drying | Enhanced stability and dissolution rate of tablets. | (Chiang et al. 2023) |
| PVPVA and Soluplus® | Nifedipine | Spray drying and hot melt extrusion | All SDs improved dissolution, but stability varied, with spray drying PVPVA-based SD showing the best overall performance. | (O’Connell et al. 2025) |
| Soluplus® | Glycyrrhetinic acid | Freeze drying | Enhanced solubility and bioavailability and anti-inflammatory effect. | (Hao Wang et al. 2022) |
| Soluplus® | Telmisartan | Hot melt extrusion | Enhanced dissolution rate and stability compared to a commercial product. | (Giri et al. 2021) |
| Soluplus® and PVP | Bosentan | Hot melt extrusion | Enhanced the dissolution rate for both carriers. Better dissolution performance with Soluplus®. | (Harish, Murthy, and Chandrasekhar 2017) |
| β-cyclodextrin, PEG 6000, PVP K30, and Eudragit® L100 | Azithromycin | Wet grinding and solvent evaporation - vacuum drying | β-Cyclodextrin SD (1:2 w/w, solvent evaporation) increased solubility 4 times over the pure drug. | (Huynh et al. 2023) |

PVP: polyvinylpyrrolidone, PVPVA: polyvinylpyrrolidone/vinyl acetate, HPMC: hydroxypropyl methylcellulose, HPMCAS: hydroxypropyl methylcellulose acetate succinate, PEG: polyethylene glycol, Tg: glass transition temperature.

In the table, 'Manufacture technique' primarily refers to the final drying step in solvent-evaporation methods for solid dispersion, particularly spray drying or freeze drying. However, these processes are preceded by essential steps, including the dissolution and mixing of components in a common or miscible solvent, as detailed in Section 3.

Table S2. Pharmaceutical solid dispersions prepared with natural polymers, highlighting the carrier, active ingredient, preparation method, and principal results.

| **Carrier** | **Active compound** | **Preparation method** | **Main results** | **Reference** |
| --- | --- | --- | --- | --- |
| Bovine serum albumin | Indometacin | Spray drying and freeze drying | Solubility was remarkably increased in the SDs with a high drug: polymer molar ratio. Freeze drying resulted in higher solubility than spray drying. | (Khoder et al. 2018) |
| Chitosan | Andrographolide | Spray drying | The active compound's solubility and dissolution rate were increased. | (Sari et al. 2019)( |
| Chitosan | Tanshinone | Freeze drying | Improved dissolution rate and bioactivity, with drug pH-responsive release. | (Luo et al. 2019) |
| Chitosan and carboxymethylchitosan | Diflunisal | Kneading and solvent evaporation - vacuum drying | Higher porosity in the chitosan SDs prepared by solvent evaporation enhanced dissolution rate, while SDs with carboxymethylchitosan led to similar outcomes regardless of preparation method. | (Lucio, Zornoza, and Martínez-Ohárriz 2022) |
| Egg white protein | Hydrochlorothiazide | Kneading | Solubility and release rates were enhanced. | (Telange et al. 2021) |
| Gelatine | Carbamazepine, cinnarizine, diazepam, itraconazole, nifedipine, indomethacin, darunavir, ritonavir, fenofibrate, griseofulvin, ketoconazole and naproxen | Freeze drying | Gelatine emerged as a suitable polymer for forming SDs, effectively converting the active compounds into completely or partially amorphous states. | (Pas, Vergauwen, and Van den Mooter 2018) |
| Isomalt, maltitol and saccharin | Griseofulvin | Melting technique - quench cooling | Both eutectic mixtures and amorphous SD improved the drug dissolution rate. | (França et al. 2022) |
| Maltodextrin | Nystatin | Solvent evaporation – oven drying | The system with a ratio of 1:6 (drug: polymer) was amorphous, improving the anti-biofilm effect of nystatin compared with the pure drug. | (Benavent, Torrado-Salmerón, and Torrado-Santiago 2021) |
| Maltodextrin and Gum Arabic | Artemisin | Freeze drying | The carriers were able to form SDs, reducing the crystallinity and maintaining the active compound’s functionality. | (Meliana et al. 2020) |
| Mung bean porous starch | Albendazole | Solvent evaporation - agitation under temperature (27 ºC) | The new starch showed the feasibility of improving the solubility and dissolution rate of the active compound. | (Nadaf, Jadhav, and Killedar 2021) |
| Sodium  alginate, guar gum, xanthan gum, and locust bean gum | Carvedilol | Kneading | SD produced with sodium alginate indicated the highest solubility and dissolution rate. | (Sopyan et al. 2023) |
| Sodium alginate | Tanshinone | Freeze drying | The SD improved the dissolution rate and oral absorption rates, besides in vivo bioavailability. | (Luo et al. 2020) |
| Sodium alginate and sodium acetate | Dexlansoprazole | Solvent evaporation - oven drying | Solubility improved 24 times compared to the pure drug. | (Gulia et al. 2023) |
| Whey protein isolate (β-lactoglobulin, α-lactalbumin, and casein glycomacropeptides) | Non-identified drugs (crystalline compounds A and B) | Dry ball milling | β-lactoglobulin and α-lactalbumin outperformed glycomacropeptides and whey protein isolate in stabilizing amorphous compound A, while β-lactoglobulin, α-lactalbumin, and whey protein isolate enhanced the dissolution rate of compound B better than glycomacropeptides. | (Leng et al. 2023) |
| Whey protein isolate and whey protein hydrolysate | Indomethacin, carvedilol and furosemide | Dry ball milling | Physical stability, solubility, and dissolution rate increased compared with pure model drugs. | (Mishra et al. 2019) |
| Xanthan, guar and acacia gums | Etoricoxib | Solvent evaporation - agitation at room temperature | The solubility and dissolution rate increased significantly. | (Sapkal et al. 2020) |
| Zein | Prednisolone | Solvent evaporation - oven drying | In general, the zein was suitable for producing SDs and improving drug bioavailability. The drug: polymer ratio of 1.3:2 resulted in a higher dissolution rate. | (Van Ngo et al. 2016)( |
| Ziziphus spina-Christi gum | Loratadine  and glimepiride | Kneading, solvent evaporation - oven drying, and the co-grinding process | All the SDs indicated improvement in drug solubility. | (Alwossabi et al. 2022) |
| β-lactoglobulin | Furosemide, indomethacin, carvedilol, and celecoxib | Spray drying | All the SDs significantly improved the solubility when compared to the pure drugs. Neutral pH favoured the interactions drug-polymer. | (Zhuo et al. 2023)) |

In the table, 'Manufacture technique' primarily refers to the final drying step of solid dispersion in solvent evaporation methods, particularly spray drying or freeze drying. However, these processes are preceded by essential steps, including the dissolution and mixing of components in a common or miscible solvent, as detailed in Section 3.

**References**

Agafonov, M., S. Ivanov, and I. Terekhova. 2021. “Improvement of Pharmacologically Relevant Properties of Methotrexate by Solid Dispersion with Pluronic F127.” *Materials Science and Engineering C* 124 (May): 112059. https://doi.org/10.1016/j.msec.2021.112059.

Aldeeb, R. A. E., M. A. E. G. Mahdy, H. M. El-Nahas, and A. A. Musallam. 2023. “Design of Mirtazapine Solid Dispersion with Different Carriers’ Systems: Optimization, in Vitro Evaluation, and Bioavailability Assessment.” *Drug Delivery and Translational Research* 13 (9): 2340–52. https://doi.org/10.1007/s13346-023-01316-9.

Alwossabi, A. M., E. S. Elamin, E. M. M. Ahmed, and M. Abdelrahman. 2022. “Solubility Enhancement of Some Poorly Soluble Drugs by Solid Dispersion Using Ziziphus Spina-Christi Gum Polymer: Solubility Enhancement of Some Poorly Soluble Drugs by Solid Dispersion.” *Saudi Pharmaceutical Journal* 30 (6): 711–25. https://doi.org/10.1016/j.jsps.2022.04.002.

Benavent, C., C. Torrado-Salmerón, and S. Torrado-Santiago. 2021. “Development of a Solid Dispersion of Nystatin with Maltodextrin as a Carrier Agent: Improvements in Antifungal Efficacy against Candida Spp. Biofilm Infections.” *Pharmaceuticals* 14 (5). https://doi.org/10.3390/ph14050397.

Biedrzycka, K., and A. Marcinkowska. 2023. “The Use of Hot Melt Extrusion to Prepare a Solid Dispersion of Ibuprofen in a Polymer Matrix.” *Polymers* 15 (13): 2912. https://doi.org/10.3390/polym15132912.

Chiang, C. W., J. W. Lubach, T. Chen, S. Chin, J. Ly, W. Zhang, H. H. Hou, and K. Nagapudi. 2023. “Development of an Amorphous Solid Dispersion Formulation for Mitigating Mechanical Instability of Crystalline Form and Improving Bioavailability for Early Phase Clinical Studies.” *Molecular Pharmaceutics* 20 (5): 2452–64. https://doi.org/10.1021/acs.molpharmaceut.2c01056.

Chivate, A., A. Garkal, K. Hariharan, and T. Mehta. 2021. “Exploring Novel Carrier for Improving Bioavailability of Itraconazole: Solid Dispersion through Hot-Melt Extrusion.” *Journal of Drug Delivery Science and Technology* 63 (June): 102541. https://doi.org/10.1016/j.jddst.2021.102541.

Elkanayati, R. M., S. Omari, A. A. A. Youssef, M. Almutairi, A. Almotairy, M. Repka, and E. A. Ashour. 2024. “Multilevel Categoric Factorial Design for Optimization of Raloxifene Hydrochloride Solid Dispersion in PVP K30 by Hot-Melt Extrusion Technology.” *Journal of Drug Delivery Science and Technology* 92 (February): 105362. https://doi.org/10.1016/j.jddst.2024.105362.

França, M. T., T. Martins Marcos, P. F. A. Costa, G. C. Bazzo, R. Nicolay Pereira, A. P. Gerola, and H. K. Stulzer. 2022. “Eutectic Mixture and Amorphous Solid Dispersion: Two Different Supersaturating Drug Delivery System Strategies to Improve Griseofulvin Release Using Saccharin.” *International Journal of Pharmaceutics* 615 (March): 121498. https://doi.org/10.1016/j.ijpharm.2022.121498.

Giri, B. R., J. Kwon, A. Q. Vo, A. M. Bhagurkar, S. Bandari, and D. W. Kim. 2021. “Hot-Melt Extruded Amorphous Solid Dispersion for Solubility, Stability, and Bioavailability Enhancement of Telmisartan.” *Pharmaceuticals* 14 (1): 1–18. https://doi.org/10.3390/ph14010073.

Gulia, R., S. Singh, S. Arora, and N. Sharma. 2023. “Development and Optimization of Hydrotropic Solid Dispersion of Dexlansoprazole Using Central Composite Design Approach.” *J. Integr. Sci. Technol* 11 (4): 559. http://pubs.thesciencein.org/jist.

Harish, R., T. E. G. K. Murthy, and K. B. Chandrasekhar. 2017. “Formulation and Evaluation of Bosentan Solid Dispersion.” *Asian Journal of Pharmaceutics* 11 (1): 75–82.

Huynh, D. T. M., H. T. Hai, N. M. Hau, H. K. Lan, T. P. Vinh, V. De Tran, and D. T. Pham. 2023. “Preparations and Characterizations of Effervescent Granules Containing Azithromycin Solid Dispersion for Children and Elder: Solubility Enhancement, Taste-Masking, and Digestive Acidic Protection.” *Heliyon* 9 (6): e16592. https://doi.org/10.1016/j.heliyon.2023.e16592.

Jarrells, T. W., X. Yuan, and E. J. Munson. 2025. “Impact of Storage Conditions on the Physical Stability of Amorphous Solid Dispersions Containing Two Structurally Similar Drugs.” *Molecular Pharmaceutics* 22 (6): 2927–38. https://doi.org/10.1021/acs.molpharmaceut.4c01279.

Khoder, M., H. Abdelkader, A. ElShaer, A. Karam, M. Najlah, and R. G. Alany. 2018. “The Use of Albumin Solid Dispersion to Enhance the Solubility of Unionizable Drugs.” *Pharmaceutical Development and Technology* 23 (7): 732–38. https://doi.org/10.1080/10837450.2017.1364267.

Kissi, E. O., R. Nilsson, L. P. Nogueira, A. Larsson, and I. Tho. 2021. “Influence of Drug Load on the Printability and Solid-State Properties of 3D-Printed Naproxen-Based Amorphous Solid Dispersion.” *Molecules* 26 (15): 4492. https://doi.org/10.3390/molecules26154492.

Leng, D., B. Bulduk, T. Widmer, O. Wiborg, M. Sanchez-Felix, and K. Löbmann. 2023. “Protein Based Amorphous Solid Dispersion: A Case Study Investigating Different Whey Proteins at High Drug Loading.” *Pharmaceutical Research* 40 (7): 1865–72. https://doi.org/10.1007/s11095-023-03542-9.

Lin, X., L. Su, N. Li, Y. Hu, G. Tang, L. Liu, H. Li, and Z. Yang. 2018. “Understanding the Mechanism of Dissolution Enhancement for Poorly Water-Soluble Drugs by Solid Dispersions Containing Eudragit® E PO.” *Journal of Drug Delivery Science and Technology* 48 (August): 328–37. https://doi.org/10.1016/j.jddst.2018.10.008.

Liu, Y., Y. Zhang, Q. Yan, X. Zhong, and C. Hu. 2024. “Evaluation of Microstructure , Dissolution Rate, and Oral Bioavailability of Paclitaxel Poloxamer 188 Solid Dispersion.” *Drug Delivery and Translational Research* 188: 329–41. https://doi.org/10.1007/s13346-023-01400-0.

Lucio, D., A. Zornoza, and M. C. Martínez-Ohárriz. 2022. “Role of Microstructure in Drug Release from Chitosan Amorphous Solid Dispersions.” *International Journal of Molecular Sciences* 23 (23): 15367. https://doi.org/10.3390/ijms232315367.

Luo, C., W. Wu, X. Lin, Y. Li, and K. Yang. 2019. “A Novel Tanshinone IIA/Chitosan Solid Dispersion: Preparation, Characterization and Cytotoxicity Evaluation.” *Journal of Drug Delivery Science and Technology* 49 (February): 260–67. https://doi.org/10.1016/j.jddst.2018.11.024.

Luo, C., W. Wu, S. Lou, S. Zhao, and K. Yang. 2020. “Improving the in Vivo Bioavailability and in Vitro Anti-Inflammatory Activity of Tanshinone IIA by Alginate Solid Dispersion.” *Journal of Drug Delivery Science and Technology* 60 (June): 101966. https://doi.org/10.1016/j.jddst.2020.101966.

Martynek, D., L. Ridvan, M. Sivén, and M. Šoóš. 2025. “Stability and Recrystallization of Amorphous Solid Dispersions Prepared by Hot-Melt Extrusion and Spray Drying.” *International Journal of Pharmaceutics* 672 (March). https://doi.org/10.1016/j.ijpharm.2025.125331.

Meliana, Y., D. Utami, M. Septiyanti, E. T. Wulandari, M. Ghozali, W. K. Restu, S. Fahmiati, and R. A. A. Lelono. 2020. “Characterization of Artemisinin Solid Dispersion in Maltodextrin and Gum Arabic by Freeze Dried and High Energy Milling Methods.” *Macromolecular Symposia* 391 (1): 1–4. https://doi.org/10.1002/masy.201900186.

Metre, S., S. Mukesh, S. K. Samal, M. Chand, and A. T. Sangamwar. 2018. “Enhanced Biopharmaceutical Performance of Rivaroxaban through Polymeric Amorphous Solid Dispersion.” *Molecular Pharmaceutics* 15 (2): 652–68. https://doi.org/10.1021/acs.molpharmaceut.7b01027.

Mishra, J., A. Bohr, T. Rades, H. Grohganz, and K. Löbmann. 2019. “Whey Proteins as Stabilizers in Amorphous Solid Dispersions.” *European Journal of Pharmaceutical Sciences* 128 (February): 144–51. https://doi.org/10.1016/j.ejps.2018.12.002.

Mora-Castaño, G., M. Millán-Jiménez, A. Niederquell, M. Schönenberger, F. Shojaie, M. Kuentz, and I. Caraballo. 2024. “Amorphous Solid Dispersion of a Binary Formulation with Felodipine and HPMC for 3D Printed Floating Tablets.” *International Journal of Pharmaceutics* 658 (June): 124215. https://doi.org/10.1016/j.ijpharm.2024.124215.

Nadaf, S., A. Jadhav, and S. Killedar. 2021. “Mung Bean (Vigna Radiata) Porous Starch for Solubility and Dissolution Enhancement of Poorly Soluble Drug by Solid Dispersion.” *International Journal of Biological Macromolecules* 167: 345–57. https://doi.org/10.1016/j.ijbiomac.2020.11.172.

Ngo, H. Van, P. K. Nguyen, T. Van Vo, W. Duan, V. T. Tran, P. H. L. Tran, and T. T. D. Tran. 2016. “Hydrophilic-Hydrophobic Polymer Blend for Modulation of Crystalline Changes and Molecular Interactions in Solid Dispersion.” *International Journal of Pharmaceutics* 513 (1–2): 148–52. https://doi.org/10.1016/j.ijpharm.2016.09.017.

O’Connell, P., J. H. Yoon, L. M. Geever, D. Kumar, A.-M. Healy, and D. R. Serrano. 2025. “Accelerated Predictive Stability (APS) Strategies Applied to Screening Pharmaceutical Formulations: A Comparison of Spray Dried and Hot Melt Extruded Nifedipine Amorphous Solid Dispersions.” *International Journal of Pharmaceutics*, October, 126012. https://doi.org/10.1016/j.ijpharm.2025.126012.

Pas, T., B. Vergauwen, and G. Van den Mooter. 2018. “Exploring the Feasibility of the Use of Biopolymers as a Carrier in the Formulation of Amorphous Solid Dispersions – Part I: Gelatin.” *International Journal of Pharmaceutics* 535 (1–2): 47–58. https://doi.org/10.1016/j.ijpharm.2017.10.050.

Sapkal, S. B., V. S. Adhao, R. R. Thenge, R. A. Darakhe, S. A. Shinde, and V. N. Shrikhande. 2020. “Formulation and Characterization of Solid Dispersions of Etoricoxib Using Natural Polymers.” *Turkish Journal of Pharmaceutical Sciences* 17 (1): 7–19. https://doi.org/10.4274/tjps.galenos.2018.04880.

Saraf, I., R. Roskar, D. Modhave, M. Brunsteiner, A. Karn, D. Neshchadin, G. Gescheidt, and A. Paudel. 2022. “Forced Solid-State Oxidation Studies of Nifedipine-PVP Amorphous Solid Dispersion.” *Molecular Pharmaceutics* 19 (2): 568–83. https://doi.org/10.1021/acs.molpharmaceut.1c00678.

Sari, R., D. Setyawan, D. Retnwati, and R. Pratiwi. 2019. “Development of Andrographolide‑chitosan Solid Dispersion System: Physical Characterization, Solubility, and Dissolution Testing.” *Asian Journal of Pharmaceutics* 5 (1): 5–9.

Sawicki, E., J. H. Beijnen, J. H. M. Schellens, and B. Nuijen. 2016. “Pharmaceutical Development of an Oral Tablet Formulation Containing a Spray Dried Amorphous Solid Dispersion of Docetaxel or Paclitaxel.” *International Journal of Pharmaceutics* 511 (2): 765–73. https://doi.org/10.1016/j.ijpharm.2016.07.068.

Shamsuddin, S., M. Fazil, S. Ansari, and J. Ali. 2016. “Atorvastatin Solid Dispersion for Bioavailability Enhancement.” *Journal of Advanced Pharmaceutical Technology and Research* 7 (1): 22–26. https://doi.org/10.4103/2231-4040.169873.

Smeets, A., R. Koekoekx, C. Clasen, and G. Van den Mooter. 2018. “Amorphous Solid Dispersions of Darunavir: Comparison between Spray Drying and Electrospraying.” *European Journal of Pharmaceutics and Biopharmaceutics* 130 (January): 96–107. https://doi.org/10.1016/j.ejpb.2018.06.021.

Sopyan, I., N. Adiningsih, S. Megantara, and S. S. Marvita. 2023. “Solubility Enhancement of Carvedilol by Solid Dispersion Technique Using Sodium Alginate, Guar Gum, Xanthan Gum, and Locust Bean Gum as Polymers.” *Indonesian Journal of Chemistry* 23 (2): 349–57. https://doi.org/10.22146/ijc.77698.

Telange, D. R., S. P. Jain, A. M. Pethe, and P. S. Kharkar. 2021. “Egg White Protein Carrier-Assisted Development of Solid Dispersion for Improved Aqueous Solubility and Permeability of Poorly Water Soluble Hydrochlorothiazide.” *AAPS PharmSciTech* 22 (3): 1–15. https://doi.org/10.1208/s12249-021-01967-2.

Wang, Hao, R. Li, Y. Rao, S. Liu, C. Hu, Y. Zhang, L. Meng, et al. 2022. “Enhancement of the Bioavailability and Anti-Inflammatory Activity of Glycyrrhetinic Acid via Novel Soluplus®—A Glycyrrhetinic Acid Solid Dispersion.” *Pharmaceutics* 14 (9). https://doi.org/10.3390/pharmaceutics14091797.

Yang, L., P. Wu, J. Xu, D. Xie, Z. Wang, Q. Wang, Y. Chen, et al. 2021. “Development of Apremilast Solid Dispersion Using TPGS and PVPVA with Enhanced Solubility and Bioavailability.” *AAPS PharmSciTech* 22 (4): 142. https://doi.org/10.1208/s12249-021-02005-x.

Zhuo, X., Z. Sener, A. Kabedev, M. Zhao, A. Arnous, D. Leng, V. Foderà, and K. Löbmann. 2023. “Mechanisms of Drug Solubility Enhancement Induced by β-Lactoglobulin-Based Amorphous Solid Dispersions.” *Molecular Pharmaceutics* 20 (10): 5206–13. https://doi.org/10.1021/acs.molpharmaceut.3c00577.
